# Care-seeking practices for non-communicable chronic conditions in a low-income neighborhood in Southern India

**DOI:** 10.1371/journal.pgph.0002074

**Published:** 2023-06-29

**Authors:** Nilanjan Bhor

**Affiliations:** Indian Institute for Human Settlements, Bengaluru, India; University College London, UNITED KINGDOM

## Abstract

**Background:**

Given that access to healthcare is less challenging in urban India, evidence shows that affordable government healthcare services are underutilized by the vulnerable and disadvantaged groups. There are emerging studies on healthcare seeking behavior in the context of short-term morbidities and communicable diseases that attempted to understand this gap of underutilization of government healthcare services, but similar studies are rare in the context of non-communicable diseases (NCDs) and associated chronic conditions. Given the urban health system is ill- prepared and ill-equipped to deliver NCD services, it is important to understand how the vulnerable and disadvantaged groups seek healthcare for chronic conditions. This article investigates the care-seeking practices of these individuals living in a low-income neighborhood and care-seeking pathways for chronic conditions.

**Methods:**

The study is conducted at Kadugondanahalli—a low-income neighborhood with the presence of a recognized slum, in Bengaluru city. A total of twenty in-depth interviews are conducted with individuals diagnosed with non-communicable chronic conditions. Participants were selected through purposive and snowball sampling method. The data is collected between January 2020 to June 2021.

**Results:**

The study participants practice a wide range of care-seeking practices based on the management of comorbidity and multimorbidity, recognizing the symptoms and severity, experiences of family members, belief, and purchase and consumption of medicines. These practices clearly highlighted not only the nuances of non-adherence to the long-term treatment and medications, but it also strongly influences the care-seeking behavior, which in turn make the care-seeking continuum very complex. The care-seeking continuum attempted each of the components (i.e. the screening, diagnosis, treatment, and control) of NCD care cascade but participants often failed to do screening on time, delayed diagnosis, and did not meet the treatment goals, leading to their conditions becoming further uncontrolled due to the care-seeking practices they practice. These practices delayed not only the diagnosis but also the completion of each component of the care cascade.

**Conclusion:**

This study emphasizes strengthening of the health system in addressing the individual and community level practices, that significantly affect the entire care-seeking continuum, in the sustained monitoring and adherence to the treatment of chronic conditions.

## 1. Introduction

In the 20^th^ century, in India, government rural healthcare centers are labeled as treatment centers for communicable diseases and maternal and child health problems [1], and this perception on the healthcare centers has continued to persist in the 21^st^ century even with the emergence of the non-communicable diseases (NCDs) epidemic. Considering a smaller number of government-provided urban healthcare centers till the implementation of the National Urban Health Mission in 2015, private healthcare providers dominated in urban areas, which positively shaped the care-seeking by the urban population. With the inception of a dedicated National NCD program in 2010, urban healthcare centers are currently equipping themselves to provide NCD services. As per the data provided by the National NCD Monitoring Survey of 2017–18 [2], NCD services, such as ambulatory facilities and facilities for NCD management, routine screening, and availability of essential technologies and medicines, are yet to be fully implemented in the urban healthcare centers at primary and secondary levels of health system in India. The question arises: does the care-seeking by utilizing government health services improve with the availability of NCD services in government urban healthcare centers? Does this change in a low-income neighborhood in ways we do not yet fully understand?

Care-seeking is a well-known concept in health literature. Existing literature has represented care-seeking as care-seeking behavior, treatment-seeking behavior, health (or healthcare)-seeking behavior and healthcare-seeking practices, but care-seeking behavior is the most used terminology. Care-seeking behavior (or any of the above-specified terminologies) is understood as actions undertaken by individuals who perceive themselves as having a health problem or being ill for the purpose of finding an appropriate remedy [3]. It is clear that health problems and illness are two different entities for which healthcare is sought. Literature on care-seeking behavior predominantly used quantitative methodology, and the parameters used to assess the behavior pattern are decision-making for seeking care at the individual/family level, choice of medical system of care (allopathic or other alternative medicines), type and sub-type of healthcare providers visited (government or private including self-care, qualified or unqualified), hospital admissions, out-of-pocket expenditure, access to healthcare providers, quality of treatment and/or satisfaction on healthcare services, referral or reasons for switching healthcare provider, and overall understanding of the care-seeking pathway for each visit to healthcare providers. The literature using qualitative methodology helped explore the why and how of these parameters. In addition, perceptions and practices were the two unique components of the qualitative research which were studied in the context of socio-cultural attitudes and beliefs. Given the parameters used, it is clear that care-seeking behavior and care-seeking practices are different concepts, but studies have likely failed to address/acknowledge this. Practices are often difficult to quantify and interpreted as articulations of various ways to achieve optimal health. These practices are mostly socio-culturally rooted and influenced by health perceptions. Care-seeking practices are the practices of individuals that are metamorphically structured and, in turn, influenced by the socially embedded practices, to define the care-seeking behavior. This further shapes the care-seeking pathway, i.e., the making of every single choice (or every single attempted path) on treatment actions to adapt depending on the available options. This interconnectedness leads to the care-seeking continuum, i.e., the care-seeking practices–behavior–pathway continuum. This care-seeking continuum influences each component of NCD care cascade, i.e., screening, diagnosis, treatment, and control [4].

Studies on care-seeking practices based on the proposed care-seeking continuum are rare, but the care-seeking behavior and pathways have been studied in the context of short-term morbidities, chronic communicable diseases and maternal and child health. With the takeover of the disease burden by NCDs in the 21^st^ century as per the global burden of disease estimates [5], there is growing literature on estimating the prevalence of NCDs, which have been mostly self-reported assessments without direct measurements, and its association with socio-economic factors, NCD risk factors, and out-of-pocket expenditure, with minimal attention given to care-seeking practices and understanding the care-seeking pathway. It could be due to the nature of chronicness, i.e., the long-term adherence to the treatment and medications for chronic diseases/conditions that pushes the financial burden on to individuals/families, with mostly the vulnerable and disadvantaged groups experiencing the worst/most severe impact on their lives. The question arises: should the care-seeking continuum for NCDs be different from short-term morbidities and communicable diseases, and how? Therefore, given the present situation of ill-preparedness of the urban health system to provide NCD services, it is important to understand the challenges faced by the vulnerable and disadvantaged groups in urban areas to utilize government health and NCD services throughout the care-seeking continuum, and how perception of health influences care-seeking for NCD services.

This study is conducted with such vulnerable and disadvantaged groups residing in a low-income neighborhood in Bengaluru. After identifying the gaps in the care-seeking literature on NCDs and associated chronic conditions, the focus of this study was set to understand the care-seeking practices practiced by the individuals residing in this neighborhood for their diagnosed chronic conditions. The understanding and acceptance of the care-seeking practices by healthcare providers in NCD services has high potential to reduce the delay in diagnosis and sustained treatment of these chronic diseases/conditions. But, before jumping to our research, we will briefly understand the care-seeking pattern in the context of short-term morbidities and communicable diseases as evident from health literature.

### 1.1 Care-seeking by low-income neighborhood

**Fig 1:**
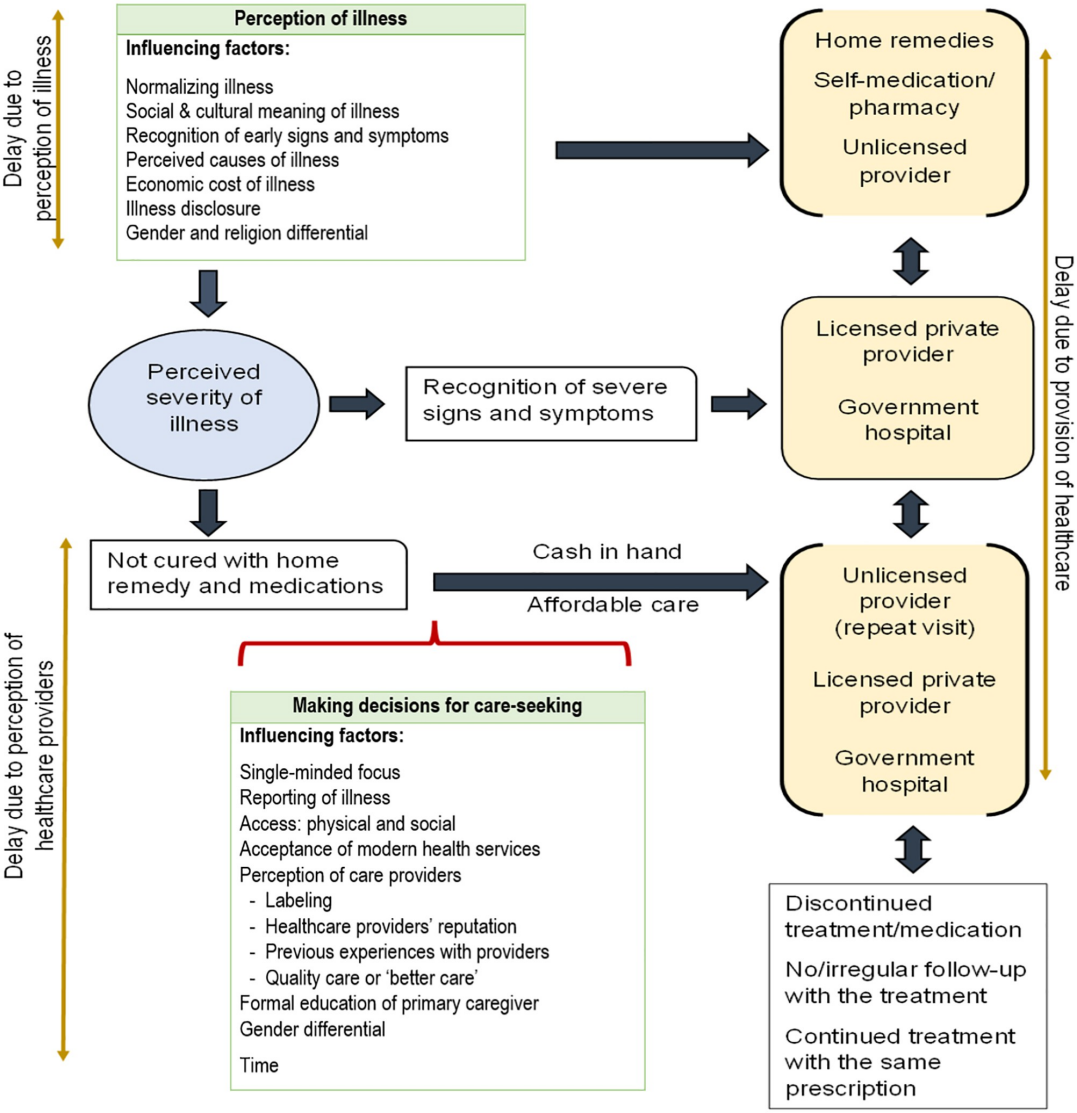
Care-seeking pathway showing factors influencing delays in care-seeking by individuals from low-income neighborhoods in India.

Fig 1 depicts the framework of the care-seeking pathway, the factors influencing delay in seeking healthcare, and the type of healthcare providers throughout the care-seeking pathway in low-income neighborhoods in India. This framework was developed by synthesizing evidence from existing literature [1, 6–38] on care-seeking behavior in low-income neighborhoods (represented as slums in the literatures). The illnesses studied, as evident from the literatures, varied from short-term morbidities (such as fever and diarrhea) to communicable diseases (HIV/AIDS, Tuberculosis, Malaria, Measles, respiratory tract infections, and acid peptic diseases). The literatures related to maternal and child health was not included in this synthesis. Due to the lack of enough literatures, an important limitation of this study, this synthesis also excluded literatures on care-seeking behavior for diabetes and hypertension (or NCDs) in low-income neighborhoods in India.

The pathway clearly revealed that care-seeking is influenced by the severity of the illness. In the pre-severity stage, the care-seeking decisions based on the perception of illness mostly led to not seeking healthcare from qualified providers, given the decisions are strongly influenced by factors that are socio-culturally rooted with mythical explanations. The normalizing factor, recognition of early signs and symptoms, economic cost of illness, and gender and religion differential were found common for all the illnesses. The healthcare choices availed at this stage are mostly the cheaper options confined to one’s knowledge on self-care and availing symptomatic medicines from pharmacies and/or unqualified/unlicensed providers without confirming the diagnosis. The delay at this stage likely happens in the provision of care due to non-referral to specialized care by these providers when repeat visits are made for similar concerns. Therefore, these socio-cultural factors in the pre-severity stage push individuals into the severity stage or to the stage of continued effort for cure. These challenges highlight the initiation of treatment at the pre-severity stage in the continuum of care model. During the severity stage, having enough knowledge on the recognition of culturally defined severe signs and symptoms of the illnesses by the individuals or caregivers has led them to seek healthcare from the qualified providers.

But effort is continued, i.e. the stage of continued effort to get cured, if not cured at the pre-severity stage and non-recognition of any severity of the illness. This is a formal care-seeking stage in which a shift from the perception of illnesses to the perception on healthcare providers is observed. Therefore, factors influencing decision-making in this stage are confined to illness reporting, a quick-recovery as a treatment outcome through better care (where better care is qualified as the ready dispensation of medicines and administration of injections), acceptance of modern healthcare services, and perception of and access to healthcare providers. Individuals enter this stage only if they fulfil the two mediating criteria: cash in hand and affordable care. There is higher chance of switching healthcare providers in this stage from the one’s opted during the pre-severity stage, but it is also evident that the switch may be to a similar type of healthcare provider, i.e., an unqualified provider, given the mediating criteria are met. The delay occurs for making decisions for the identified factors by the individuals, and delay in diagnosis and referral are caused by delay in healthcare service provision. This formal care-seeking stage highlights the challenges of continued monitoring and adherence in the continuum of care model.

Therefore, similar to short-term morbidities and communicable diseases, it is important to understand the care-seeking continuum in the context of NCDs. The question arises: how complex and different is the care-seeking continuum for NCDs compared to short-term morbidities and communicable diseases?

## 2. Methodology

### 2.1 Design

Phenomenology [39] was applied in a qualitative study using in-depth interviews. The study conducted with individuals diagnosed with non-communicable chronic conditions, i.e., hypertension and diabetes and also residing in Kadugondanahalli (KG Halli), a low-income neighborhood, in Bengaluru, India. This paper is focused on the care-seeking practices of the two selected chronic conditions. Given the rareness of health equity research in the context of NCDs, the findings of this study positively add value to the current knowledge and promote research on the care-seeking continuum in the context of NCDs and associated chronic conditions. In this study, we adhere to the guidelines of the Consolidated criteria for reporting qualitative research (COREQ) checklist [40].

### 2.2 Setting

KG Halli (Ward No. 30) was purposefully selected as the field site of PEAK Urban health research implemented between 2019 and 2021. It is one of the 198 administrative units of Bengaluru city, the capital of Karnataka state. It has a population of more than 34,842 individuals spread over 0.7 square kilometers, including an area classified as a recognized slum which possesses characteristics such as lack of access to improved water, improved sanitation, sufficient living space, durability of housing and secure tenure [41]. It was observed that the local municipal government provided housing to many families of this slum and construction of buildings continues to accommodate many more. The population in KG Halli comprises and forms a mixed social and economic neighborhood with people from socio-economically backward to lower-middle-class families representing all major religions of the country. While conducting interviews, participants reflected that the “majority of the families living here [at KG Halli] migrated from the neighboring southern states long ago”.

Previous surveys at KG Halli [13] assessed the burden and health-seeking for non-communicable chronic conditions between 2009–2010 and 2012–2013. These surveys reported that hypertension and diabetes were the two most commonly reported conditions, and health-seeking from government healthcare centers was reduced by 8% and 9% for hypertension and diabetes, respectively, between 2010 to 2012. There is one government primary healthcare center (PHC) and one community healthcare center (CHC) within a walkable (1 kilometer) distance. The National Programme for Prevention & Control of Cancer, Diabetes, Cardiovascular Diseases & Stroke was implemented in this CHC, and therefore, both the NCD clinic and counselling services are available and functioning. The PHC and CHC are located next to each other. There is a Pradhan Mantri Bhartiya Jan Aushadhi Kendra (pharmacy shop) located within the CHC premises near the entrance gate, but outside the hospital building, that provides generic quality medicines with 30%–70% discount with prescriptions and sometimes without prescriptions to follow-up patients. In both the PHC and CHC, there is a list of drugs displayed at the entrance which shows the name of the drugs under each category of illness. Therefore, the question arises: why have NCD services from government health facilities declined or are under-utilized by the vulnerable and disadvantaged groups in KG Halli, as reported by previous studies? The current study was designed to respond to this question (and the questions posed earlier in this article) by identifying the various care-seeking practices of these groups.

In addition to well-equipped government healthcare centers for NCD care, the presence of a mix of private healthcare centers ranging from clinics to nursing homes, specialty hospitals in and around the neighborhood, and a medical college hospital within a distance of 1 km from government primary/community healthcare centers, which provide either primary, specialist or in-patient care or both makes the health system in KG Halli highly complex. A high number of diabetic clinics, pharmacy shops, and diagnostic centers, independent or attached with the healthcare providers/hospitals, are also present in this mixed healthcare system. Therefore, it is clear that the availability of government and private services and accessibility to those services is not a challenge at KG Halli.

### 2.3 Participant selection

Participants were recruited using a purposive and snowball sampling method. The procedure involved door-to-door visits and selecting eligible individuals who met the inclusion criteria for participation in the interview. The inclusion criteria were: individuals must be diagnosed with either diabetes or hypertension or both, be residing at KG Halli, and provide consent to participate.

A total of 20 recruited participants, of aged above 40 years, who participated in the interviews fall under three categories: (i) self-reported and diagnosed (this refers to a chronic condition that has been self-reported by individuals and has also been confirmed by a health service provider); (ii) diagnosed and currently under treatment (this refers to individuals who are receiving some form of treatment (including medications) whether diagnosed or prescribed or self-care); and (iii) diagnosed but currently without treatment (this refers to individuals who are diagnosed but discontinued treatment (including medications)). The majority of the participants have been diagnosed and are under treatment, except one found to have discontinued treatment and medications during the time of the interview.

### 2.4 Data collection

The rapport building in the community was started from June 2019 and actual data collection was done between January 2020 to June 2021 using two methods: direct observations in the community and in-depth interviews. A total of 20 in-depth interviews were conducted. Informed verbal consent including consent for audio recording was obtained for voluntary participation in the study. The average duration of each interview was about 30 minutes. Interviews were recorded using an audio recorder. Interviews were conducted in Hindi, English, Tamil and Telugu. Interview guide was once tested during the rapport building. Two researchers (the author and another researcher from the same affiliated institution) were involved in the data collection to address this muti-lingual barriers.

An unstructured interview guide was developed to conduct the interviews, which was improved based on information that emerged in the course of data collection. However, the overarching issues in the interview guides focused on understanding illness experiences before and after diagnosis with hypertension and diabetes, in the context of extracting information on care-seeking practices and challenges faced in care-seeking and long-term treatment adherence for these two conditions. All the interviews held face-to-face. For few interviews, one family member was present with the participant during the interview.

### 2.5 Data analysis

After completion of 20 interviews, the data recorded using the audio recorder was processed by the researchers in the following way: transcripts were prepared in the same (local) language the interviews were conducted, i.e., mostly in Hindi, Tamil or Telugu, or sometimes in mixed languages, and then translated into English. Interviews conducted in English were directly transcribed into English. Next, multiple readings of the transcripts were performed to generate codes and recognize the broader themes originating from the codes by using ATLAS.ti9 software. Then, the narratives under each theme were analyzed to identify those narratives strongly associated with the themes. Field notes from direct observations, collected after each interview, were also reviewed along with the themes.

### 2.6 Ethical considerations

Institutional ethics approval was obtained from Indian Institute for Human Settlements to conduct the research at KG Halli. Further informed verbal consent was obtained from each participant, who have given their autonomous decision on willingness to participate, before commencement of each interview and, at the same time, the process also entailed explaining the study information such as purpose and objectives of the research, confidentiality aspects, and rights to participate and withdraw from the interview. Verbal consent was documented using audio recorder where participant’s identity i.e., the names of the participants were replaced with unique identity numbers, and hence, participants names were confidential during the transcription and translation activities. Due to confidentiality, the study participant’s names are also not being used in this paper, and represented as ‘P’. Participants names and other essential details were documented in the field notes for record keeping during data collection.

### 2.7 Limitations of this study

This study conducted in a neighborhood with specific characteristics. Other neighborhoods in India may or may not processes similar characteristics. Further this study applied qualitative method and limit the associated sample size. Therefore, extrapolation of the findings should be done carefully.

## 3. Results

The care-seeking continuum followed by the participants throughout the NCD care cascade were found to be complex. The complexities of practices in turn affected the behavior towards treatment choices, and repeated switching from one healthcare center to another made the pathway even complex. It was difficult to develop the care-seeking pathway for every move participants made from the onset of the symptoms to the last visit to the healthcare centers for NCD care. The behavior towards treatment choices is not limited to the easy availability and accessibility to healthcare providers in KG Halli. A Few participants even visited healthcare centers beyond KG Halli to find appropriate healthcare providers for their treatment. Five themes, as explained below, have been created, which clearly highlight the nuances of the care-seeking practices.

### (i) Care-seeking based on management of comorbidity and multimorbidity

In addition to either diabetes or hypertension or both, participants were found to have one or more comorbid and multimorbidity conditions such as asthma, thyroid, cholesterol, stroke, and musculoskeletal and eye problems. In the majority of cases, the diagnosis and care-seeking of a comorbid condition was the main reason the participants got tested and further diagnosed with hypertension and diabetes (also referred to as BP (blood pressure) and sugar, respectively, in the participants’ responses). This is why the participants did not seek care in the first place, leading to continued delay in screening and diagnosis and further in initiating early treatment.

*Initially I was having pain on one side of my body*. *My colleagues suggested I see a doctor*. *I went to the doctor and found that I have cholesterol*. *The doctor asked me whether I eat more non-vegetarian food*. *I said*, *yes*, *I like it*. *The doctor also asked me if I have diabetes*. *I said*, *I have not checked*. *Then I did the sugar test and was found to be diabetic*.

(P6)

*I came to know that I have BP almost 5 years ago when my treatment was going on for chikungunya*.

(P10)

Sometimes, participants were accidentally diagnosed with BP and/or sugar as healthcare providers failed to recognize the appropriate symptoms on time. In a few cases, the doctor only prescribed symptomatic medicine rather than identifying the problem through laboratory tests, and multiple referral/shifting to another health provider for service dissatisfaction caused panic to the patient. Participant P7 expressed, “*I fell down [fainted] twice*. *One time it happened in the toilet*. *That time I received only first aid and BP was not measured*. *There was giddiness*, *sweating*, *leg pain*, *etc*. *Further I met a doctor at Bowring hospital and they found that I have BP*. *Then I met the doctor in a nearby clinic and he found that I have thyroid*. *I have reported my leg pain*, *but no one diagnose the problem correctly*. *They just give some tablet and send me to St*. *John’s hospital saying they can’t treat this here … that’s it*.”

Participant P4 expressed that hypertension levels had increased due to diarrhea, “*I went to a private hospital to treat my diarrhea and started on prescribed tablets*. *After taking those tablets also I was looking very dull and weak as told to me by my neighbor*. *Then my husband took me to our family doctor (at a clinic)*. *The doctor said that there is no diarrhea and I have high BP…that they could not treat it there*, *and to go to Bowring hospital [government tertiary care hospital]*. *At Bowring hospital*, *they found that I have both BP and sugar*. *The doctor didn’t check me properly in the private hospital and that’s why my BP increased due to diarrhea*.”

### (ii) Care-seeking based on recognizing the symptoms and severity

Most of the participants do remember the initial symptoms that led them to a diagnosis of either hypertension or diabetes or both. Giddiness and headache were the most commonly expressed symptoms of hypertension, along with burning and vomiting sensation, and body pain (especially in the leg) for diabetes. Disturbed sleep and changes in sleeping pattern are common for both hypertension and diabetes. Yet, realizing the presence of symptoms did not lead participants to go for check-ups immediately before the diagnosis. But after the diagnosis, the majority of the participants expressed that they continued to experience these symptoms and it led to an immediate visit to the doctor. Care-seeking practices practiced arond recognizing the symptoms and severity positively delayed the screening and early diagnosis and further initiation of treatment for hypertension and diabetes. Participant P9 stated, “*I used to have pain in my hand*, *having difficulty in eating food*, *slowly the pain started in another hand too*, *didn’t work out after massaging*. *The pain increased*. *Then*, *one of the family members told me that I have sugar*, *and to go and get it checked*. *Then*, *after testing*, *I came to know that I have sugar*. *I was doing tailoring work*. *I used to feel a lot of discomfort*. *Slowly my leg also started hurting; so I discontinued*.”

*My head started spinning*. *I used to get very tired*. *I couldn’t walk*. *I used to get thirsty for water*. *Something was happening to my body from the inside*. *I did not know what was happening to me and I thought somebody must have done something to me*. *I was so weak*. *I was telling myself to have faith in Allah*. *Later a person took me to the hospital for a check-up*. *I was fat then*. *After my son died*, *I had become fat*. *So*, *I went for a check-up*. *So*, *then when the doctor finished the tests*, *I asked him what was wrong with me and he told me that I have sugar*. *He said my sugar level was very high*.

(P11)

*I used to visit our family doctor often for my recurring knee pain*. *I used to take injections for pain relief*. *He then advised me to go to a government hospital and get myself checked*. *That is when I was told that I had sugar*.

(P8)

Recognition of the early symptoms does not mean immediate care-seeking will occur, especially before the diagnosis.

*I had these symptoms for about two months but didn’t go to the doctor due to other health problems like itching eyes*, *which was causing by dust and work-related commitments*. *Then my sister told me that I shouldn’t have giddiness … and I should go to the doctor immediately and check my BP*. *I went to doctor and started taking tablets*. *I hadn’t taken it seriously at the beginning*.

(P3)

Cultural traditional practices, mostly learnt from family members, used as home remedies by a few participants to relive the initial symptoms led to delay in screening and diagnosis, and initiation of treatment. Participant P4 said, “*Initially when I was getting chest discomfort*, *burning sensation in leg and feet*, *and tiredness*, *I used home remedies like keeping the feet in warm water*, *keeping banana peel on feet and eating lots of cold items*. *But I didn’t get relief*. *Then I did a blood test in a laboratory and was found to be diabetic as per the laboratory personnel*. *I have taken the report to the doctor and he has given advice on diet and walking*.” P5 said, “*I had lot of leg pain when I started preparing and consuming the power drinks using fenugreek leaves*, *Ome*, *etc*. *… My leg pain and tension were reduced*. *I have been consuming this drink for the last three … no*, *two years*. *After drinking*, *you can take tablets also*. *I suggest others to use this remedy too*.”

On asking how they came to know that their BP and/or sugar is uncontrolled, participant P1 said, “*Suddenly my legs shook and I felt very tired*. *I visited the doctor immediately for a BP check-up and came to know that my BP had increased*. *… I measure BP once in a year; otherwise*, *I continue BP tablets daily*.” P6 said, “*I feel giddiness when my sugar level is low or high*. *Then*, *I visit doctor immediately*.” P7 said, “*When I have headache*, *I know that my BP has increased*.” P8 said, “*Dental problems happen when my sugar level increases*.” P10 said, “*When I have sudden pain in my eyes*, *I know something is wrong*. *If there is increase [in BP levels]*, *then I take care of my diet by avoiding salty and spicy food*. *In a couple of days or so my BP comes back to normal*.”

There are many other short- or long-term illnesses that may appear due to diagnosis with and management of these chronic conditions, as claimed by many participants, which increases the burden of illness on these individuals and worsens their financial conditions, as P8 said, “*Expenses were less*, *earlier when I had no dental problems*, *but had only sugar and BP*.”

A few participants have taken steps to secure their lives based on the predicted future struggles after realizing the severe impact of the diagnosis of hypertension and diabetes on their life.

*It did take me a week*. *During that [before diagnosis] time*, *I was alone*. *I didn’t have a family*. *I got married after this [diagnosis] happened*. *I got married four years ago*. *I was alone till then*. *My family members said that my health is not good now*, *and I don’t have anyone to take care of me*. *That is when I thought of getting married*. *Till then I hadn’t thought about it*. *I was living alone*. *I would eat at the hotel or sometimes my friends would get me food*. *I was eating well and living my life*. *I had a washing machine*. *I would wash my clothes and get them ironed*. *Life was going on like that*. *I got married after I got diabetes*.

(P10)

### (iii) Care-seeking based on experiences of family members

It was found that care-seeking towards adherence to the treatment and medications for hypertension and diabetes is difficult for those families where both the participant and their spouse have been diagnosed with one or both of these conditions. Participant P3 who feels that she was diagnosed with hypertension and diabetes due to having managed her husband’s condition, said,

*My husband who was a watchman has BP*. *Sometimes it was normal*, *and sometimes high [based on self-perception on health]*. *He used to take tablets when it was high but said he had no need of tablets when it was normal*. *Suddenly he got a stroke*. *Due to financial problems after having taken him to three different hospitals*, *we have stopped going*. *Now he is partially paralyzed … unable to do anything since the last two years*, *unable to speak*, *hear and move hands*. *Before*, *he took care of me very well*. *Currently*, *I am getting free medicines for both BP and sugar*, *but it is very difficult to get medicines and nutrition syrup prescribed by the doctor to my husband as we do not have the money*. *It costs about Rs*. *700 to Rs*. *800 in the medical shop*.

Participant P4 was able to recognize her symptoms early because she was taking care of her mother-in-law who was diagnosed with diabetes.

*She [mother-in-law] shared with me that if I get diabetes then I will have symptoms like burning sensation*, *itching*, *giddiness*, *disturbance in sleep*, *etc*. *She also shared that if anyone in our family has BP or sugar*, *then it will happen to us [children]*. *I also took her many times to Bowring hospital and there the doctor asked if anyone had sugar in our family*.

Not seeking care was found to be a common practice.

*My father had a heart attack and passed away*. *He used to say every day that he was having more pain in legs and joint pains*. *He put his leg in hot water or rice water to reduce the pain and to continue his work*. *He did not stop going to work*. *He also didn’t once go to the hospital and he used to take some tablet*. *My mother too had BP and sugar*, *and had a stroke and passed away almost 15 years ago*. *Currently*, *my husband does not take any tablets for any health issues*. *He just goes to the clinic when he feels feverish and takes an injection … that’s it*.

(P7)

Advice from family members and extended family after experiencing unexpected physical discomfort to go for a check-up for BP and/or sugar was shown to positively influence care-seeking.

*My elder brother told me that you should check BP and sugar after 45 [years of age]*. *That’s why I went to a doctor and got tested*. *I came to know then that I have both BP and sugar*. *The doctor said that medicine will not work and I have to take insulin for sugar*. *Now I feel good after taking insulin*.

(P9)

### (iv) Care-seeking based on belief

Acceptance of the diagnosis has immensely affected care-seeking practices. Participant P11 said that she did not accept the diagnosis of diabetes initially, “*I told him [the doctor] I had no sugar or anything as I had not done anything to get it*, *and God would keep me safe and I was just weak because I kept thinking about my son’s death*. *He told me I had sugar*. *He gave me the medicines*.” Even though participants were aware that they might get BP or sugar and changed their behavior towards diet, they accepted their diagnosis as god’s wish. P11 said, “*My mother has sugar*. *My aunty (mother’s elder sister)*, *who already passed away*, *used to tell us that we too would get sugar*. *So*, *I too didn’t have sweets of any kind*. *I was eating very plain sugarless food*, *even sugarless tea*. *But Allah wanted it to happen to me*.”

Participants are well connected with their peers (such as friends, neighbors etc.) diagnosed with BP and/or sugar through social networking. P4 expressed, “*Many of them [peers] passed away due to sugar*. *A sugar patient will live for around 20 to 25 years*, *that’s it*”. Advice from their social network positively affected care-seeking practices, and led to delay in initiation of treatment. Participant P2 said,

*I didn’t accept the diagnosis [of diabetes] initially and went to do the blood test in another laboratory*. *But it said I was diabetic in that report as well*. *This time*, *someone told me if I start on tablets for sugar then I will have to continue taking it for life*, *rather I could control my sugar by changing my food habits like avoiding non-veg*, *rice*, *consuming less salt and sugar*, *etc*. *I did not take any tablets for about two to three months and started eating only ragi ball and chapatti with some curry*. *Further I can control food at home*, *but not outside while meeting friends*. *By doing so*, *I lost weight*, *my eyes became very dull*, *everyone started asking me what happened to me*. *Again*, *I did a blood test at Ananda laboratory in Shivajinagar and took the report to ESI hospital*. *Here*, *the doctor explained to me how I should take the tablet*. *Now I will not miss my tablets and ‘it’s become a habit like brushing our teeth*.*’ Now*, *I am confident that sugar can’t be controlled by changing food habits and it is a lie*. *I advise sugar patients to take tablets and not try to control it by changing food habits … when you are in the initial stage*, *the dosage will be less*, *easier to control and you should not reach the injection [insulin] stage*.

It is clear here how the perception of managing diabetes by changing food habits has been shifted to dependency on medicines, whereas, in reality, both are important for the management of chronic conditions. This belief is leading to harm and causes significant delay in care-seeking. This is why regular follow-up treatment and clear advice from treating doctors regarding diet and consumption of tablets become important to overcome these beliefs. P3 said, “*I meet the doctor once in two or three months*. *Both the doctor and the nurse advise doing exercise*, *what to eat and what not*, *and give medicines*. *Such advice is given mainly by the doctors who speak our language*. *Other English doctors just check the pulse and prescribe medicines*.” But the follow-up treatment is also affected by the participant’s financial status, as P4 said, “*Yes*, *I will go for check-up once a month but I will do the sugar test only if I have money*, *otherwise not*.”

Further, beliefs around side-effects of medicines are also contribute to stoppage or irregular intake of medicines.

*I was not going for a check-up every month*. *So*, *I thought to go and get the blood test done once*. *This time my wife did an experiment with water…*. *Then she said I was diabetic*, *and not to take sugar tablet as it will affect my kidney*. *Thereafter my blood test report was normal and we decided to stop tablets*. *But the earlier experience was stuck in my head that I should not stop tablets*. *One day we both went to the government hospital and the doctor said to continue the same tablet*. *We bought those and came back home*.

P(11)

### (v) Care-seeking based on purchase and consumption of medicines

Non-adherence to medications is frequently practiced by the study participants, influenced by the following practices. Participants buy medicines from various sources and through various ways to manage the continuity of the treatment for their particular conditions. Participant P1 said, “*Sometimes*, *when I have leg and back pain*, *I want to go to the government hospital*. *But my daughter is a government employee and she would give me one strip of tablet for these problems*, *and rest I buy from the medical shop*”. P2 added,

*I used to get facilities from my company (where I used to work) to procure sugar tablets from the Employees State Insurance (ESI) Hospitals under the insurance scheme*. *But I lost that job due to COVID*. *Then I went to the KG Halli government hospital and they have given the same tablet of another brand which I was borrowing from ESI*. *Simultaneously*, *the doctor here also checked my BP and increased the dose from 2*.*5mg to 5mg*. *Earlier*, *the BP tablet was prescribed by the doctor in a clinic that I visit for any health problems near my house*.

The perception of allopathic versus ayurvedic medicines positively influences decision on care-seeking.

*Now that my sugar level is normal*, *I am thinking of replacing allopathic medicines with ayurvedic*, *as allopathic is not good for health*. *I am also losing my memory power which I think is because of consuming these tablets*. *The advertisement for ayurvedic medicines comes on television*. *I didn’t get any difficulty even after taking tablets of other brands*. *But I have to go to the clinic when I fall sick and then I will have a problem as the doctor there prescribes allopathic medicines*. *So*, *I am thinking of continuing with both*.

(P2)

*He [the doctor] told me I have sugar and gave medicines*. *At that time*, *I didn’t have money*. *I was taking traditional medicine [ayurvedic medicine] and things were very bad*. *I was getting breathless at times*. *My hands and legs would get sweaty*. *My son’s friend [who passed away] got me treated*. *Then they told me that they [a nearby hospital] were giving medicines for sugar and we should go there and get it*. *They would give it for free*. *So*, *we went there and bought the medicine*. *Since then*, *I have been taking the tablets*.

(P11)

In the case of those participants receiving free/cheap medicines from Sarvagna hospital (also referred to by participants as George hospital), which runs on public–private partnership, located in the slum, follow-up treatment is provided each time the doctor prescribes the next slot of medicines. Participant P3 said “*When I go to collect tablets after I have completed the previous slot for a month or two*, *doctors check BP and sugar*. *They don’t give tablets without doing this*.” But this was not the case for the participants who receive treatment and medications from other healthcare centers.

However, most of the participants receiving free medicines had to buy or manage medicines from other sources because they do not receive enough medicines for the month due to lack of medicine stock. P9 said, “*I get free/cheap medicines from Sarvagna hospital including injections*. *If at the time there are no tablets for a month or no stock*, *they suggest buying them from outside [from medical shops]*.” P8 had also faced a similar situation.

*Once the BP tablets were less in number*. *I did not take it for five days*. *When I went and asked at the hospital pharmacy*, *they shouted at me*, *saying why I had not taken it*. *They did not give me more*. *They don’t give it for free nor do they give it for money*. *They don’t give it in the middle of the month*. *We have our book*. *They give medicines according to the book*. *They give it every month*. *If you ask in between*, *they don’t give the tablets*. *As per their rules they are not supposed to*. *Even if we are late by a day*, *they don’t give it to us*. *That’s how it is*. *I went and asked at the medical store [pharmacy outside the hospital] and they gave me one with a different name*. *I did not take it*. *My BP was high and I had not taken the tablets and at the hospital they said not to do that again*. *It increased by twenty points and I took extra tablets*. *I had not taken the tablets for three days*. *They told me not to do it again*.

Buying and managing medicines from other sources varied with the participants’ financial situation. P4 said, “*My daughter gets to buy free medicines of Rs*.*1000 through the church and gives medicines to me*. *She also gets cheaper medicines from Ambedkar hospital*. *The family doctor helps by providing the prescription in my daughter’s name*.”

In a few cases, it was found that the choice of health facilities is different for care-seeking for follow-up treatment, and to procure medicine. Almost all participants visit family doctors. Practitioners who are referred to as “family doctors” by the participants are family physicians who have a clinic and practice in the study area. P8 said, “*I take medicine from George hospital as well as buy it from medical shops*. *Last week I went to our family doctor and checked my BP and sugar*.” P10 said, “*We go to the family doctor just behind our house*. *I bring tablets and injection from there [medical shop attached to the clinic]*. *It reduces a little bit and then it becomes normal*. *He takes less money from us*. *If he takes Rs 100 from everyone*, *then he takes about Rs 80 Or 50 From us*. *There are times when I don’t have money for his fees*, *but he doesn’t mind it*. *He tells me to pay the next time*. *A visit to the clinic is sufficient most of the time for us*. *We don’t go to hospitals as such for consultations*.”

Further, the choice of selecting a healthcare center for *c*are-seeking and follow-up treatment is not only influenced by access to free/affordable medicines but also the suitability of those medicines. Therefore, participants were switching between pharmacies to test and recognize the suitable medicines for themselves.

*I went to a nearby doctor [at a clinic near her house] for BP*, *to our family doctor*. *His prescribed medicines were not suited to control my BP*. *Then I went to Sherifa hospital and saw a BP doctor*. *After taking medicines from that doctor*, *my BP became normal*. *I have also gone to a private hospital for sugar*.

(P9)

*I don’t go to the George Hospital*. *I got high sugar because of this hospital*. *My sugar level had reached 400 to 500*. *They have given me health card also through which I get tablets free of cost*. *They say free*, *but there are charges for consultation and tests [laboratory tests]*. *That’s why I have stopped going there*. *… I am good after taking private hospital medicines*.

(P6)

*After taking those tablets [taken from the government hospital]*, *it [sugar] had gone up*. *Those tablets didn’t have an effect*. *It didn’t work for me*.

(P11)

*I was taking BP tablets from Bowring hospital as one of my neighbors took me there saying they provide good medicines*. *But I was feeling more giddiness and was not able to walk properly after taking these tablets*. *I would walk like an alcoholic person*. *So*, *I have stopped taking medicines from there and shifted to Sarvagna hospital from where I am currently taking sugar treatment and tablets*.

(P3)

Participants search for alternative treatment, by knowing the side effects of medicines for themselves, where healthcare providers/doctors understand their concerns and prescribe tablets accordingly.

*I am allergic to tablets*. *I get gastric problem…earlier I got urine infection too*. *Afterwards*, *I had to get admitted [in the hospital]*. *There*, *the doctor said that I have gastric problems related to blood and to not take tablets*. *That’s why the doctor also gives insulin injection for my sugar and does not give high dose tablets*. *I get free treatment and medicines including injections from Sarvagna hospital*, *so I go there once every month*.

(P9)

*I had aches and pains*, *probably some issues with my nerves*. *So*, *the doctor has prescribed this medicine [participant took out the medicine from the medicine box and showed to the interviewer] to me*. *He said that it doesn’t have side effects and is safe to consume*. *I have been taking this medicine for the last five months*.

(P10)

The effectiveness of medicines is spoken about by the majority of the participants. P8 said, “*Even after taking injection [indicated to insulin] my energy is low*.” But this may be affected by the diet and self-management practices followed for those medicines: as expressed by P5 “*10 mg is to be taken from that vial and a maximum of 15 mg if required*. *If I overeat or eat biriyani or something heavy like that*, *then an extra 5mg is taken*. *Then everything comes up to normal*.” P11 added, “*I have to buy only the injection for my sugar*. *Nothing happens with the injection*. *So*, *I just take the tablets and manage … No*, *I don’t have a fridge to store it [insulin bottle]*. *I store it in water*. *There is small mud pot*. *I keep it in that*. *This is the poor person’s fridge*. *I am keeping it like this since two years and I have not told the doctor about this*.” P9 said,

*I went to doctor and asked why my sugar is not in control even after following proper diet and tablets*. *The doctor asked how I was taking the injection [insulin]*? *I said*, *I remove the bottle 15 minutes before from the fridge and then take it*. *The doctor told me to take the injection within 10 minutes and keep the bottle immediately in the fridge; otherwise it would not be effective much to control my sugar*. *After following this procedure*, *my sugar was controlled*.

Participant P1 stated the practice of self-prescription of medicines for symptoms like leg pain, tiredness, and headache based on the experiences of previous visits made to the doctor, “*After I come back home from seeing the doctor*, *I write down in my notebook…for what health problem I went to the doctor*, *what medicines were prescribed*, *etc*. *The next time when I feel I have a similar health problem*, *I buy the same medicines as written in the book from the medical shop and take it*.” But this participant has also clearly expressed following of the doctor’s advice for not stopping medicines for BP and thyroid even if it is normal.

## 4. Discussion

It is clear from the results that the study participants practice a wide range of care-seeking practices that influence care-seeking behavior and treatment adherence for hypertension and diabetes. It is clear from the results that all the study participants have reached each step of the NCD care cascade, i.e., screening, diagnosis, treatment, and control of both hypertension and diabetes care. The care-seeking continuum (care-seeking practices–behavior–pathway continuum) influenced each of these components of NCD care cascade, but participants often failed to do screening on time, delayed diagnosis, and did not meet the treatment goals, leading to their conditions becoming further uncontrolled due to the identified practices. These practices delayed not only the diagnosis but also the completion of each component of the care cascade. As Banerjee and colleagues [42] have reported, “the poor control of BP, despite a large proportion of patients being on antihypertensive medications, needs explanation.” This is clearly explained by our study participants through their various practices, as specified in the results. Therefore, uncontrolled hypertension and uncontrolled diabetes, which alternate between controlled and uncontrolled states, were often reported by the study participants throughout the care-seeking continuum. The study conducted by Dey et.al. [43] found similar socio-demographic and behavioral findings but this study also identified the health system barriers that affecting screening, diagnosis and treatment of diabetes and hypertension. The care-seeking pattern that emerged from this study is explained below.

First, screening for hypertension and diabetes is affected due to the weak outreach system by the government primary or community healthcare centers, as evident from the direct observations made throughout the data collection period. In addition, all the participants, except one, also reported that they had not attended any such outreach camps. A few participants reported of a door-to-door screening camp conducted by a nearby church and by the trust hospital located in the neighborhood. Second, a significant delay was caused by the participants due to home remedies and traditional cultural practices, self-prescription of medicines and purchasing symptomatic medicines from pharmacies, and sometimes due to neglecting physical discomfort for mostly behavioral and economic reasons. The study conducted by Mendenhall and colleagues [25] in the capital of India, Delhi, revealed that self-care practices were found significant in treating diabetes: such as some form of modified diet, walking, yoga, eating sadabahar, neem, and fenugreek leaves, and seeds of java plum, etc. Mendenhall reported that these practices led to normalizing health which severely delays diagnosis and treatment. Not being able to overcome physical discomfort, recognizing the severity of the discomfort due to decreased productivity, and emergency events like fainting spells have led participants to visit a doctor. However, there has been significant delay caused by healthcare providers too, mostly by prescription of symptom-specific medicines especially for diabetes, and not prescribing clinical testing due to the treatment of other confirmed diagnosed conditions. Almost all participants reported visiting the family doctor, as the first point of contact, for any discomfort and other health problems. But in severe cases, decisions were also made by a few participants to visit private hospitals for advanced treatment. It is clear that these practices severely delayed diagnosis of hypertension and diabetes, and that is why the majority of the study participants were accidentally diagnosed with hypertension and/or diabetes when tests for these were done randomly while visiting a doctor for severe physical discomfort or another confirmed diagnosis. A few participants also reported that blood testing for diabetes prescribed by the doctor to confirm the diagnosis is not done immediately for economic reasons. Advice from family members and extended family was also shown to positively influence care-seeking towards hypertension and diabetes on realizing symptoms, mostly influenced by the knowledge transferred by older family members regarding various symptoms that led to hypertension and diabetes, screening for hypertension and diabetes at the age of 45 years, and lessons from accompanying with their family members to the doctor.

After being diagnosed with hypertension and/or diabetes, delays occurred in initiation of treatment in the case of a few study participants. The practices influencing initiation of treatment were mostly socio-culturally rooted, and led to non-acceptance of the diagnosis: attributing the diagnosis to ‘karma’ and ‘god’s wish,’ traditional practices to confirm the diagnosis and not initiating medication, and re-confirming the blood test report by visiting another diagnostic laboratory. Further, the influence of their social network especially peer advice towards behavior changes with respect to diet intake influenced their decision to not initiate medication as once started it has to be continued for life. However, realizing the continued severity of the physical discomfort led to visiting the doctor (either the same doctor who confirmed the diagnosis or the family doctor) again or initiating the prescribed medications from the last visit, but it had promoted dependency on medications and giving less importance to behavior changes in diet and physical activity.

After initiating treatment, adherence to treatment and medications was the major challenge faced by the study participants. The majority of the participants expressed that they adhere to the treatment and medications, but this conclusion is influenced by their perception of health. Therefore, their diseased state does not always lead to perception of ill health and in the majority of the cases being ‘healthy’ during illness paves the way for non-adherence to or irregular treatment and medications. This could also be a reason for participants’ physical discomfort continuing after the diagnosis due to the uncontrolled state of hypertension and/or diabetes. In the control stage, participants knew of the connection between the type of the discomfort and the physiological imbalances through the controlled and uncontrolled states of hypertension and diabetes (e.g., headache leads to increased hypertension, dental problem leads to increased blood sugar level, etc.). Practices that influence non-adherence were also related to purchase and consumption of medicines. Participants choose the type of healthcare provider based on availability of affordable medicines, suitability (or acceptability) of the type of medicines, and availability of all the medicines (in terms of quantity and number of days) as prescribed by the doctor from one source. Participants purchased medicines from pharmacies of different kinds such as medical shops, medical shops attached to clinics, pharmacies in government primary/community/tertiary healthcare centers, pharmacies in private hospitals/nursing homes, Pradhan Mantri Bhartiya Jan Aushadhi Kendra (a government program which provide medicines at 30–70% discount), and trust hospitals. The occurrences of switching between pharmacies are more frequent as compared to switching between healthcare centers as the majority of the participants reported that medicines were not suitable to them. Unsuitability of the medicines was explained by the participants as those medicines that are ineffective in controlling their hypertension and diabetes based on self-perception of health, and led to feeling lethargic, walking like an alcoholic person, and suffering from more giddiness. In addition to suitability, reduced efficacy of the medicines was reported by a few participants as they continued to have low energy, found their blood sugar being uncontrolled even after taking proper tablets and diet, and suffered continuing discomfort. Some reported replacing insulin injections with tablets (without doctors’ advice) as they had observed no improvement. In few cases where participants were in insulin treatment, it was found that the reporting of inefficacious nature of insulin depends on how insulin are being stored and usage of it. In cases where participants had to purchase medicines from outside pharmacies as enough stock of medicines was not available in hospital pharmacies, especially in government healthcare centers and trust hospital providing free or cheap medicines, instead of purchasing medicines from other pharmacies, a few participants sometimes managed their medicines by using those of another family member, mostly their spouse, who had been diagnosed with a similar condition, to complete their course of medicines before the next visit to the doctor and/or purchasing the next course of medicines. A few participants reported that they willingly do not continue their medication regularly or continue medication regularly (with few irregular occurrences) but do not regularly follow the diet advice. Participants also reported using old prescriptions to purchase medicines, indicative of non-adherence to treatment and medications. Evidence [18] suggest that decrease in monthly household expenditure and dissatisfaction from modification of treatment were associated with higher use of old prescription. Therefore, though the treatment and control appear to be an integrated approach in adhering to treatment and medications, both must be investigated separately while understanding care-seeking for NCD care: treatment is focused on practices that participants follow to check their hypertension and/or diabetes levels through direct measurements in healthcare centers, and the control is focused on the practices related to medications and behavior change aspects which strongly contribute to the controlled and uncontrolled state of hypertension and diabetes. The behavioral aspect attached with medicine intake and maintaining behavior change towards diet and physical activity is strongly influenced by self-perception of health that leads to non-adherence to treatment and medications, therefore frequent fluctuation between controlled and uncontrolled states of hypertension and diabetes was common among the study participants.

The majority of the participants visited private healthcare providers, ranging from clinics to nursing homes, and secondary to tertiary care hospitals, for their follow-up treatment and medications, making the care-seeking pathway complex. Those who visited public tertiary hospitals, which are likely farther away from KG Halli, were getting subsidized and suitable medicines, and procurement of medicines was the only reason for care-seeking from government healthcare centers. Even if the participants have their preferred healthcare centers for hypertension and diabetes treatment, they simultaneously visit family doctors too for these treatments including other health problems. Therefore, it is clear that availability of and affordable NCD care in the nearby government primary and community healthcare centers is under-utilized by the study participants.

In conclusion, this study emphasizes the need for considering the care-seeking practices, in improving individualistic treatment outcomes, that significantly affect the entire care-seeking continuum for hypertension and diabetes in low-income neighborhoods. This study proposes that the health system must be strengthened towards addressing multi-morbidity management of non-communicable chronic conditions through an integrated approach, family-centered approach in addressing sustained monitoring and adherence to the NCD treatment, addressing the affordability and suitability of medicines for NCD care, and identifying local solutions to improve care-seeking practices such as strengthening outreach services and effective coverage. Further, this study also emphasizes the need for more research on care-seeking practices using the proposed care-seeking continuum for improving NCD care and services to the urban vulnerable and disadvantaged groups.
